# Sodium Tanshinone IIA Sulfonate Ameliorates Injury-Induced Oxidative Stress and Intervertebral Disc Degeneration in Rats by Inhibiting p38 MAPK Signaling Pathway

**DOI:** 10.1155/2021/5556122

**Published:** 2021-05-25

**Authors:** Shouqian Dai, Xiu Shi, Rongqing Qin, Xing Zhang, Feng Xu, Huilin Yang

**Affiliations:** ^1^Orthopedic Institute, Department of Orthopedic Surgery, The First Affiliated Hospital, Soochow University, Suzhou, Jiangsu, China; ^2^Department of Emergency Medicine, The First Affiliated Hospital, Soochow University, Suzhou, Jiangsu, China; ^3^Department of Obstetrics and Gynecology, The First Affiliated Hospital, Soochow University, Suzhou, Jiangsu, China; ^4^Department of Spinal Surgery, Gaoyou Hospital Affiliated Soochow University, Gaoyou, Jiangsu, China; ^5^Department of Orthopedics, Gaoyou People's Hospital, Gaoyou, Jiangsu, China

## Abstract

**Objective:**

Sodium tanshinone IIA sulfonate (STS) is a water-soluble derivative of tanshinone IIA, a representative traditional Chinese medicine. The aim of the study was to investigate the capability of STS to reverse injury-induced intervertebral disc degeneration (IDD) and explore the potential mechanisms.

**Methods:**

Forty adult rats were randomly allocated into groups (control, IDD, STS10, and STS20). An IDD model was established by puncturing the Co8-9 disc using a needle. Rats in the STS groups were administered STS by daily intraperitoneal injection (10 or 20 mg/kg body weight) while rats in the control and IDD groups received the same quantity of normal saline. After four weeks, the entire spine from each rat was scanned for X-ray and MRI analysis. Each Co8-9 IVD underwent histological analysis (H&E, Safranin-O Fast green, and alcian blue staining). A tissue was analyzed by immunohistochemical (IHC) staining to determine the expression levels of collagen II (COL2), aggrecan, matrix metalloproteinase-3/13 (MMP-3/13), interleukin-1*β* (IL-1*β*), IL-6, and tumor necrosis factor-*α* (TNF-*α*). Levels of oxidative stress were measured using an ELISA while activity of the p38 MAPK pathway was assessed using Western blot analysis.

**Results:**

Compared with the control group, needle puncture significantly decreased IVD volume and T-2 weighted MR signal intensity, confirming disc degeneration. These alterations were significantly attenuated by treatment with 10 or 20 mg/kg STS. Lower COL2 and aggrecan and higher MMP-3/13, IL-1*β*, IL-6, and TNF-*α* levels in the IDD group were substantially reversed by STS. In addition, treatment with STS increased antioxidative enzyme activity and decreased levels of oxidative stress induced by needle puncture. Furthermore, STS inhibited the p38 MAPK pathway in the rat model of IDD.

**Conclusions:**

STS ameliorated injury-induced intervertebral disc degeneration and displayed anti-inflammatory and antioxidative properties in a rat model of IDD, possibly via inhibition of the p38 MAPK signaling pathway.

## 1. Introduction

Intervertebral disc degeneration (IDD) is recognized as among the most common causes of lower back pain [1]. IDD is prevalent in both the middle-aged and elderly population, patients becoming increasingly younger over recent years. The condition has become an economic and healthcare burden, requiring considerable medical resources and causing substantial economic pressures [2]. Risk factors for IDD include genetics, bacteria, and viruses [3], sex [4], obesity [5], smoking [6], aging [7], and mechanical loading [8]. Pathological changes in degenerated intervertebral discs (IVDs) include narrowing of the intervertebral space, dysfunction of nucleus pulposus cells (NPCs), degradation of the extracellular matrix (ECM), rupture of the annulus fibrosus (AF), and calcification of the vertebral endplates [9]. However, the precise etiology and pathogenesis of IDD remain unclear. An increasing number of studies have concluded that IDD arises due to oxidative stress [10–12].

A previous research study demonstrated that oxidative stress at least partly leading to the structural failure of degenerated IVDs results from the excessive production of reactive oxygen species (ROS) [13]. A number of oxidative stress markers have been found and identified in degenerated human IVDs [14, 15]. ROS has been shown capable of promoting the progression of apoptosis in NPCs *in vitro* [11]. In addition, a number of external stimuli, including mechanical overloading, deprivation of nutrition, and inflammatory cytokines, can result in the accumulation of intracellular ROS [16, 17]. The observations above suggest that oxidative stress may be closely related to IDD.

The dried root of Salvia miltiorrhiza Bunge (Danshen) has multiple therapeutic properties relevant for a variety of cardiovascular diseases [18]. Sodium tanshinone IIA sulfonate (STS) is a water-soluble derivative of tanshinone IIA extracted from Danshen. Previous studies have demonstrated that STS has anti-inflammatory, antioxidative, and antiapoptotic properties [19–21]. However, until now, few studies have reported the effect of STS on an injury-induced rat model of IDD, the underlying mechanism of the possible protective actions of STS in IDD remaining largely unknown.

The present research employed an injury-induced rat model to ascertain the effect of STS on IDD. Previous evidence has shown that activation of the p38 MAPK signaling pathway is involved in oxidative stress [11, 22], and so this signaling pathway is a potential target for IDD in rats. The present study is the first to evaluate the effects of STS on oxidative stress and IDD in rats. Furthermore, the research explored the possible mechanism by which STS ameliorated the progression of IDD. The data demonstrated that STS ameliorated injury-induced oxidative stress and intervertebral disc degeneration in rats by inhibition of the p38 MAPK signaling pathway.

## 2. Materials and Methods

### 2.1. Reagents and Materials

STS (purity 99%) was obtained from Sigma-Aldrich Inc. (St. Louis, MO, USA). Enzyme-linked immunosorbent assay (ELISA) kits for superoxide dismutase (SOD, S0087), glutathione peroxidase (GSH-Px, S0056), catalase (CAT, P3541-100 ml), and malondialdehyde (MDA, S0131M) were acquired from Beyotime Biotechnology Co. Ltd. (Shanghai, China). All primary antibodies used in the study were purchased from Cell Signaling Technology, Inc. (MA, US), while secondary antibodies were from Beyotime Biotechnology Co. Ltd. (Shanghai, China). Sigma-Aldrich Inc. (St. Louis, MO, USA) supplied all other reagents.

### 2.2. Animals

Forty adult male Sprague-Dawley rats (weighing 270-320 g, 12 weeks of age) were purchased from the Animal Center at Soochow University (Suzhou, China). Animals were maintained within normal conditions and randomly allocated into four equal groups prior to surgery. All rats received various treatments, as appropriate, for two weeks and then subjected to experimental surgery, as described below. All experimental procedures were approved by the Animal Care and Experiment Committee of Soochow University (2020 Approval No. ECSU-2020000108).

### 2.3. Grouping

The experimental rats were randomly divided into four groups (10 rats in each group) as follows: (1) control (sham) group; (2) IDD group; (3) STS10 group (IDD+STS 10 mg/kg body weight); and (4) STS20 group (IDD+STS 20 mg/kg body weight). Rats in the STS groups received daily intraperitoneal injections of STS (10 or 20 mg/kg body weight) while rats in the control and IDD groups were injected intraperitoneally with the same volume of normal saline until the end of the study, at which point the rats were sacrificed. The procedures used in the present study are detailed in a previously published study [23]. The therapeutic doses were selected from those established in another study [24].

### 2.4. Rat Model of IDD

Percutaneous needle puncture has been demonstrated to be an effective method of induction of disc degeneration [25]. Following acclimatization, animals were anesthetized by inhalation of 2% fluothane in oxygen/nitrous oxide. The surgical procedure was performed on the vertebrae in the tail, as described previously [26]. In the IDD and IDD+STS groups, the Co8-9 discs were punctured using a 20-gauge needle. Sham surgery was performed on rats in the control group. Following surgery, STS or normal saline was administered to each animal once per day for four consecutive weeks, after which their tails were scanned using X-rays and MRI while being anesthetized with isoflurane and the Co8-9 discs were harvested for subsequent experiments. All experimental steps complied with the Animal Research Reporting of In vivo Experiments (ARRIVE) guidelines.

### 2.5. Radiographic Analysis and Magnetic Resonance Imaging (MRI) Scanning

After four weeks of induction of IDD, each animal was scanned with X-rays and by MRI. X-ray images were obtained using a digital X-ray machine (Shimadzu, Japan) and stored digitally. Using a previously reported method [27], the disc height index (DHI) was calculated from the mean of measurements obtained from the anterior, middle, and posterior portions of the disc which was divided by the mean height of the adjacent vertebral body using ImageJ image analysis software. Changes in DHI were recorded as %DHI and normalized to the DHI of the preoperative IVD (%DHI = DHI postsurgery/DHI presurgery∗100).

T2 mapping of MRI signal intensity is commonly used to measure the degree of IDD. The procedure was conducted as previously described [28], in a 1.5T MRI scanner (GE, USA). Briefly, all rats were scanned and the T2 signal intensity of the Co8-9 discs was recorded. The ratio of T2 signal intensity of each injured disc to the control disc was recorded from analysis using ImageJ software. Therefore, normalized IVD intensity had values ranging from 0 to 1.

### 2.6. Histological Evaluation

All harvested IVDs were fixed in 10% formalin and embedded in paraffin. Five *μ*m serial sections were obtained from the midsagittal region and stained with hematoxylin and eosin (H&E), Safranin-O Fast green, and alcian blue in order that histological changes in the IVDs could be identified and assessed using a previously described scale, providing scores from 5 to 15 points, representing IVDs that were normal to severely degenerated [26].

### 2.7. Immunohistochemical (IHC) Analysis

The disc tissue of the Co8-9 IVDs was obtained from experimental rats, as described previously [29]. Immunohistochemical staining was performed on decalcified sections, as described previously [30]. Paraffin-embedded sections (5 *μ*m) were first subjected to H_2_O_2_ for fifteen minutes and then blocked in regular blocking solution for half an hour at 37°C. The sections were subsequently incubated with rabbit primary antibodies: ECM-associated proteins collagen II (COL2, 1 : 500) and aggrecan (1 : 500), matrix metalloproteinase-3 (MMP-3, 1 : 500), MMP-13 (1 : 500), and the inflammatory factors interleukin-1*β* (IL-1*β*, 1 : 500), IL-6 (1 : 500), and tumor necrosis factor-*α* (TNF-*α*, 1 : 500) or control rabbit IgG (1 : 200 in 5% BSA), respectively, overnight at 4°C. After washing three times, the sections were then incubated with diaminobenzidine- (DAB-) based peroxidase-conjugated goat anti-rabbit secondary antibody (1 : 200) for one hour at 37°C. All images were acquired with a light microscope (Olympus, Japan) at 40x magnification. IHC staining results were then analyzed semiquantitatively using a method described previously [30]. The number of positively stained cells and staining intensity were used in scoring; the two scores were multiplied together to reflect the degree of protein expression. Images from all sections were obtained and analyzed independently by two observers that were blinded to the experimental details.

### 2.8. Enzyme-Linked Immunosorbent Assays (ELISAs)

Four common indicators of oxidative stress, SOD, GSH-Px, CAT, and MDA were quantified using the corresponding assay kit (Beyotime Biotechnology Co. Ltd., Shanghai, China) in accordance with the manufacturer's instructions. Briefly, the disc tissue was first lysed with 0.25% trypsin for 15 minutes and then centrifuged for 15 minutes at 4°C at 900 g. The supernatants and standards were added to cuvettes, and values of OD at 530 nm were recorded. The quantity of enzyme able to transform 1 mmol substrate in 1 minute was defined as 1 unit of enzyme activity. The activity of SOD, GSH-Px, CAT, and levels of MDA were calculated by reference to a standard curve.

### 2.9. Western Blot Analysis

Expression levels of p38 and p-p38 in the discs were measured using routine Western blot analysis. All disc tissues were homogenized using RIPA lysis buffer containing protease inhibitor to obtain a preparation of total protein. Equal quantities of protein were separated using routine sodium dodecyl sulfate polyacrylamide gel electrophoresis (SDS-PAGE), then transferred to polyvinylidene difluoride (PVDF) membranes. After blocking in 5% nonfat milk, the membranes were blotted with primary antibodies at 4°C overnight: p38 MAPK (1 : 1000), phosphorylated p38 MAPK (p-p38, 1 : 1000), and GAPDH (1 : 2000), then an HRP-conjugated secondary antibody at 37°C for one hour. Protein bands were then visualized using an ECL imaging system. The signal intensity of each blot was analyzed using ImageJ software. Finally, the relative expression levels of p-p38 were normalized to those of p38.

### 2.10. Statistical Analysis

All experimental data are presented as the means ± standard deviation (SD). One-way analysis of variance (ANOVA) was used to compare multiple groups after verification of normality with post hoc comparisons using a least-squares difference (LSD) method. *P* values of differences that were less than 0.05 were considered statistically significant.

## 3. Results

### 3.1. STS Reduces Narrowing of the IVD Space and Decreased T2-Weighted MRI Signal Intensity in a Rat Model of IDD

**Figure 1:**
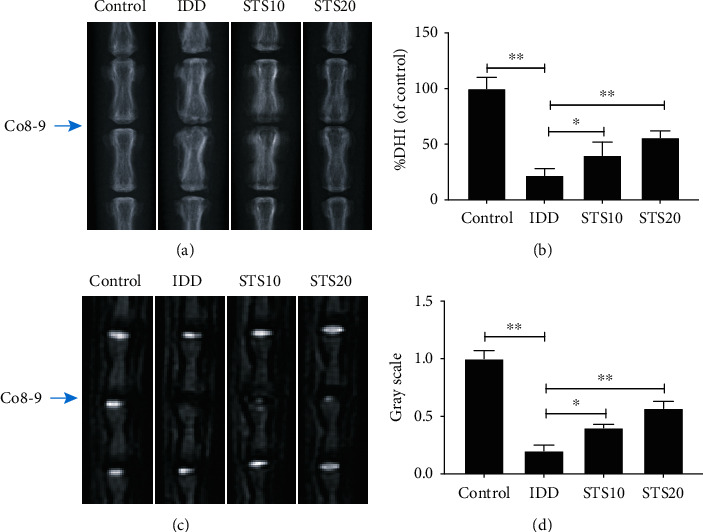
Imaging characteristics of IVDs in each group after 4 weeks. (a) Representative radiographic images of the Co8-9 discs in each group and (b) their semiquantitative analysis. (c) Representative images of T2- weighted MR in each group and (d) their semiquantitative analysis. The height of the IVD space and the signal intensity of T2-weighted MRI in the IDD group were found to be significantly lower than those of the control group. However, this decrease was substantially suppressed by treatment with 10 or 20 mg/kg STS. IDD: intervertebral disc degeneration; DHI: disc height index; STS10/20: 10/20 mg/kg sodium tanshinone IIA sulfonate; ^∗^*P* < 0.05 and ^∗∗^*P* < 0.01 compared with the IDD group (*n* = 10 per group).

Analysis of the IVD space or height was conducted and recorded as the %DHI (ratio of DHI 4 weeks after surgery to before surgery). The T2-weighted MRI signals of IVDs reflect the extent of their degeneration. Representative X-ray and T2-weighted MRI images are displayed in Figures 1(a) and 1(c). By quantitative analysis, the height of the IVD space and the signal intensity of T2-weighted MRI in the IDD group were found to be significantly lower than those of the control group (*P* < 0.01). However, this decrease was substantially suppressed by treatment with 10 or 20 mg/kg STS (*P* < 0.05 and *P* < 0.01, respectively). These imaging data demonstrate that STS can ameliorate IDD.

### 3.2. STS Inhibits the Extent of IVD Degeneration in Injury-Induced IDD Rats

**Figure 2:**
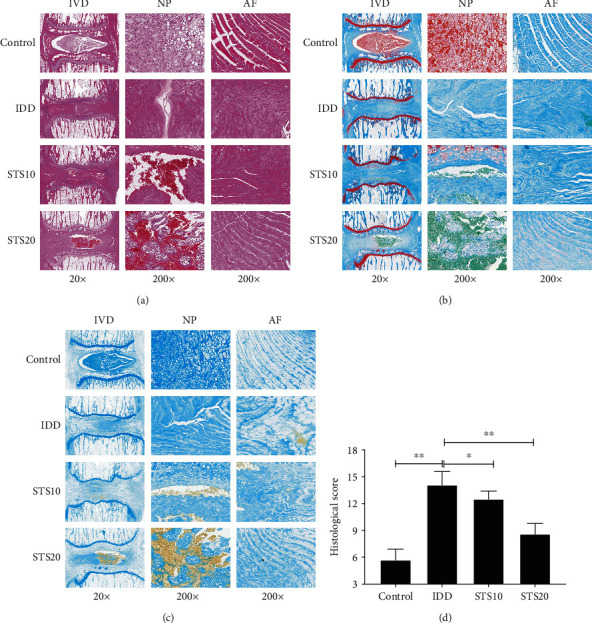
Histological analysis of IVDs in each group after 4 weeks. Representative histological images of IVDs stained with H&E (a), Safranin-O Fast green (b), and alcian blue (c) in each group. (d) Semiquantitative analysis of histological staining. Normal IVDs in the control group consisted of round NPs, distributed evenly, and integrated among collagen lamellae. Degenerated discs in the IDD groups exhibited NPs of significantly reduced size, with a clearly blurred boundary between the NP and AF. Four weeks' treatment with STS significantly rescued such disc degeneration in a dose-dependent manner. IVD: intervertebral disc degeneration; NP: nucleus pulposus; AF: annulus fibrosus; IDD: intervertebral disc degeneration; STS10/20: 10/20 mg/kg sodium tanshinone IIA sulfonate; ^∗^*P* < 0.05 and ^∗∗^*P* < 0.01 compared with the IDD group (*n* = 10 per group).

Normal IVDs in the control group consisted of round NPs, distributed evenly, and integrated among collagen lamellae, as displayed in Figure 2. Degenerated discs in the IDD groups exhibited NPs of significantly reduced size (including their complete disappearance), with a clearly blurred boundary between the NP and AF. Furthermore, disorganized inner collagen layers of the AF were bulging inward. However, four weeks' treatment with STS significantly rescued such disc degeneration in a dose-dependent manner. Semiquantitative analysis indicated that the histological scores of the IDD rats were significantly higher than those of control rats (*P* < 0.01, Figure 2). Histological scores were significantly lower in the STS10 and STS20 groups compared with the IDD group (*P* < 0.05 and *P* < 0.01, respectively). These findings demonstrated that STS ameliorates the histopathological degeneration of IVDs.

### 3.3. STS Inhibits the Degradation of COL2 and Aggrecan in the IDD Model

**Figure 3:**
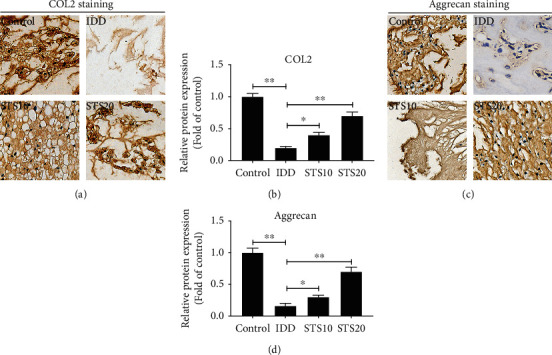
Immunohistochemical analysis of COL2 and aggrecan. (a) Representative IHC images of COL2 staining. (b) Semiquantitative analysis of COL2. (c) Representative images of aggrecan staining. (d) Semiquantitative analysis of aggrecan. Compared with the control group, IDD rats exhibited ECM with significantly less COL2 and aggrecan expression levels. This decrease was significantly restored by STS treatment indicating that STS ameliorated IDD by inhibition of ECM degradation. All images were acquired at 400x magnification. COL2: collagen II; IDD: intervertebral disc degeneration; STS10/20: 10/20 mg/kg sodium tanshinone IIA sulfonate; ^∗^*P* < 0.05 and ^∗∗^*P* < 0.01 compared with the IDD group (*n* = 10 per group).

Compared with the control group, IDD rats exhibited ECM with significantly less COL2 and aggrecan expression levels, indicating that needle puncture induced significant degradation of COL2 and aggrecan (*P* < 0.01, Figure 3). This decrease was significantly restored by STS treatment compared with that of the IDD group. The results indicate that STS ameliorated IDD by inhibition of ECM degradation.

### 3.4. STS Inhibits the Protein Expression Levels of MMP-3 and MMP-13

**Figure 4:**
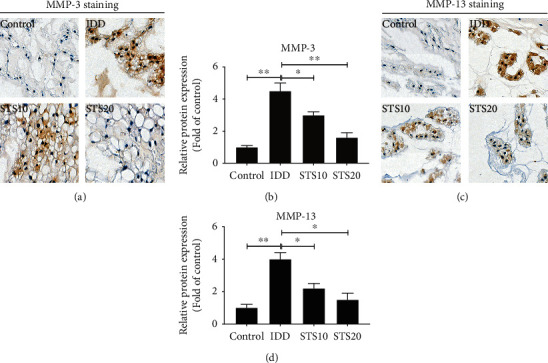
Immunohistochemical analysis of MMP-3 and MMP-13. (a) Representative IHC images of MMP-3 staining. (b) Semiquantitative analysis of MMP-3. (c) Representative IHC images of MMP-13 staining. (d) Semiquantitative analysis of MMP-13. Needle puncture in the IDD groups resulted in significant elevation of both MMP-3 and MMP-13 expressions in comparison with the control group. Treatment with STS resulted in a considerably decrease compared with the IDD group in a dose-dependent manner, indicating that STS ameliorates IDD via regulation of the activation of MMPs. All images were acquired at 400x magnification. MMP: matrix metalloproteinase; IDD: intervertebral disc degeneration; STS10/20: 10/20 mg/kg sodium tanshinone IIA sulfonate; ^∗^*P* < 0.05 and ^∗∗^*P* < 0.01 compared with the IDD group (*n* = 10 per group).

MMPs (predominantly MMP-3 and MMP-13) have been proposed as the principal catabolic enzymes in degenerated IVDs [31]. As shown in Figure 4, IVDs in the control group displayed extremely little expression of either MMP-3 or MMP-13. Needle puncture in the IDD groups resulted in significant elevation of both MMP-3 and MMP-13 expressions in comparison with the control group (*P* < 0.01). Treatment with STS (10 and 20 mg/kg) resulted in a considerable decrease compared with the IDD group in a dose-dependent manner. These data suggest that STS ameliorates IDD via regulation of the activation of MMPs.

### 3.5. STS Suppresses the Expression Levels of Inflammatory Factors in the IDD Model

**Figure 5:**
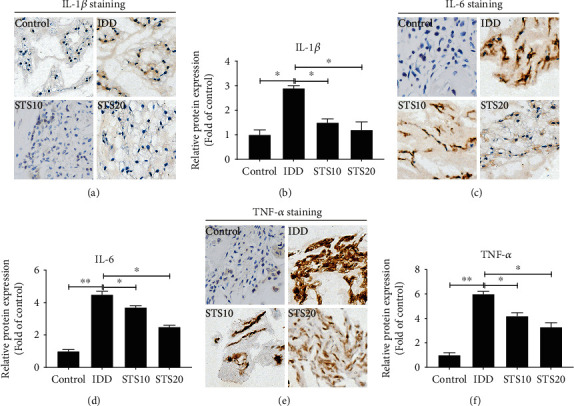
Immunohistochemical analysis of IL-1*β*, IL-6 and TNF-*α*. (a) Representative IHC images of IL-1*β* staining. (b) Semiquantitative analysis of IL-1*β*. (c) Representative IHC images of IL-6 staining. (d) Semiquantitative analysis of IL-6. (e) Representative IHC images of TNF-*α* staining. (f) Semiquantitative analysis of TNF-*α*. The expression levels of the inflammatory factors IL-1*β*, IL-6, and TNF-*α* were significantly higher in the IDD group than in the control group. However, treatment with 10 or 20 mg/kg STS significantly inhibited IL-1*β*, IL-6, and TNF-*α* levels in comparison, indicating that STS suppresses the expression levels of inflammatory factors in the IDD model. All images were acquired at 400x magnification. IL: interleukin; TNF: tumor necrosis factor; IDD: intervertebral disc degeneration; STS10/20: 10/20 mg/kg sodium tanshinone IIA sulfonate; ^∗^*P* < 0.05 and ^∗∗^*P* < 0.01 compared with the IDD group (*n* = 10 per group).

Analysis by IHC indicated that the expression levels of the inflammatory factors IL-1*β*, IL-6, and TNF-*α* were significantly higher in the IDD group than in the control group (*P* < 0.01, Figure 4). However, treatment with 10 or 20 mg/kg STS significantly inhibited IL-1*β*, IL-6, and TNF-*α* levels in comparison (*P* < 0.05, Figure 5). These data demonstrate that STS ameliorates IDD via inhibition of the production of inflammatory factors.

### 3.6. STS Regulates the Antioxidant System and Lipid Peroxidation

**Figure 6:**
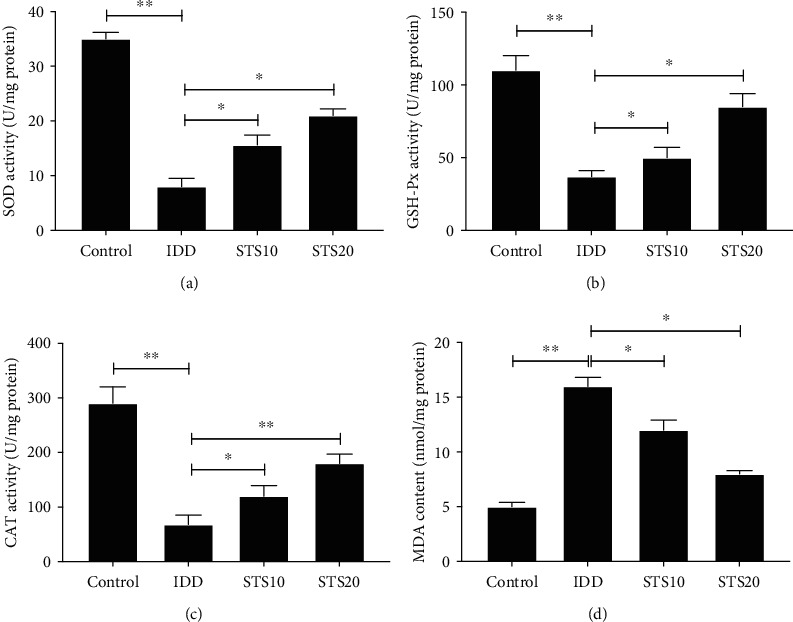
SOD, GSH-Px, CAT, and MDA contents in rat IVDs of each group. STS treatment increased the activity of (a) SOD, (b) GSH-Px, and (c) CAT that had been lowered by puncture injury and significantly decreased MDA levels (d) compared with the IDD group. These results demonstrated that STS regulates the antioxidant system and lipid peroxidation. SOD: superoxide dismutase; GSH-Px: glutathione peroxidase; CAT: catalase; MDA: malondialdehyde; IDD: intervertebral disc degeneration; STS10/20: 10/20 mg/kg sodium tanshinone IIA sulfonate; ^∗^*P* < 0.05 and ^∗∗^*P* < 0.01 compared with the IDD group (*n* = 10 per group).

As depicted in Figures 6(a)–6(c), there was a considerable decline in SOD, GSH-Px, and CAT activity in rat IVDs in the IDD group compared with control rats (*P* < 0.01). Notably, treatment with STS upregulated the activity of these components of the enzymatic antioxidant defense system in a dose-dependent manner. Moreover, the IVDs of IDD rats displayed a significantly higher MDA concentration compared with those of the control group (Figure 6(d), *P* < 0.01). However, treatment with STS (both 10 and 20 mg/kg) significantly reversed this difference. These results indicate that STS ameliorates IDD via the regulation of oxidative stress.

### 3.7. STS Regulates the p38 MAPK Signaling Pathway in the Rat Model of IDD

**Figure 7:**
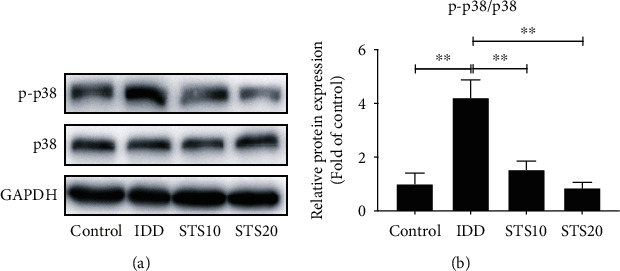
Western blot analysis of p38 and p-p38 protein expressions in each group. Activation of p-p38 MAPK was shown in IVDs of the IDD group (a). Overexpression of p-p38 in the IDD group was significantly inhibited by STS treatment (b). These data indicate that STS regulates the p38 MAPK signaling pathway in the model of IDD. GAPDH: glyceraldehyde-3-phosphate dehydrogenase; IDD: intervertebral disc degeneration; STS10/20: 10/20 mg/kg sodium tanshinone IIA sulfonate; ^∗^*P* < 0.05 and ^∗∗^*P* < 0.01 compared with the IDD group (*n* = 10 per group).

The p38 protein is known to transduce apoptotic or death signals in NPCs. Oxidative stress leads to activation and phosphorylation of the p38 signaling pathway [32, 33]. Western blot analysis demonstrated that protein expression levels of p-p38 MAPK in the IDD group were substantially higher than those in the control group (*P* < 0.01, Figure 7), indicating activation of the p38 pathway, while protein expression levels of GAPDH and p38 did not apparently change. Treatment with STS significantly downregulated p38 kinase phosphorylation compared with the IDD group, without influencing the total expression of p38 protein (*P* < 0.05, Figure 7). These data indicate that STS ameliorates IDD by inhibition of the p38 MAPK signaling pathway.

## 4. Discussion

IVD disease, the most common cause of lower back pain, is characterized by the progressive loss of ECM and a concomitant decrease in water content, changes in the structure of the IVD, and disc dysfunction. The imbalance between anabolic and catabolic processes, leading to the upregulated production of MMPs and loss of collagen and proteoglycans, finally results in alterations to the mechanical properties of IVDs and their herniation. Current research has demonstrated that STS can preserve the water content and volume of an IVD, suppressing the excessive degradation of ECM-associated proteins and inhibition of injury-induced oxidative stress in a rat model of IDD. The results of the present study indicate that STS may be a novel therapeutic agent for IDD.

As a representative traditional medicine, tanshinone IIA displays multiple pharmacological functions, but its poor water solubility has greatly restricted its further development [34]. STS, a chemically modified form of Tan IIA, exhibits considerably increased water solubility [35], with multiple pharmacological properties, including being antioxidative, anti-inflammatory, anticancer, and antiapoptotic. In the present study, we first evaluated the effects of STS on the histopathological changes within a rat puncture model of IDD. The results of the present study indicate that STS can protect IVD height and water content by reducing and inhibiting the overproduction of MMP-3 and MMP-13, which are responsible for degradation of the ECM-associated proteins COL2 and aggrecan, thus suppressing the progression of IDD. The ECM of the NP primarily consists of three components: collagen, proteoglycans, and water, of which COL2 and aggrecan are the two principal components [36]. COL2 provides the elastic strength of an IVD while aggrecan maintains the water content of the NP. MMPs participate in the degradation of ECM components, including proteoglycans and collagen. An imbalance in the synthesis and catabolism of COL2 and aggrecan leads to the onset of IDD. Previous studies have indicated that MMP-3 and MMP-13 are initially upregulated in an injured AF or NP [37, 38]. IHC staining has demonstrated that the elevated production of MMPs correlates with the progression of IDD [39]. The findings of the present study reveal that STS reduces the degradation of COL2 and aggrecan through inhibition of MMP activity, ultimately ameliorating the progression of IDD.

It has been established that IDD is mediated by the excessive production of inflammatory cytokines secreted by different IVD cells, resulting in the apoptosis and autophagy of NPCs [40–42]. Oestrogen can decrease IVD cell apoptosis and inhibit IDD in multiple ways, including the inhibition of the inflammatory cytokines IL-1*β* and TNF-*α*, reducing catabolism because of inhibition of matrix metalloproteinases, decreasing oxidative damage [43]. Previous studies have revealed that inflammation is the key event during the progression of IDD [44]. Of the various proinflammatory mediators, IL-1*β*, IL-6, and TNF-*α* are probably the most important. Increased expression levels of IL-1 and TNF have been observed in degenerated and herniated IVDs [9, 45]. In the present study, needle puncture was used to significantly increase the production of IL-1*β*, IL-6, and TNF-*α*, demonstrating that environmental stressor injury can result in inflammatory events. Treatment with STS significantly reduced the indicators of inflammation in a rat model of IDD. It has been reported that STS can reduce the expression of the inflammatory cytokines IL-6 and TNF-*α* in a mouse model of atherosclerosis [46]. STS was also found to downregulate the expression levels of IL-1*β*, TGF-*β*, and TNF-*α* via the inhibition of NF-*κ*B phosphorylation in the nucleus [47, 48]. In addition, STS has been shown to suppress the release of IL-6 and TNF-*α* in a mouse model of sepsis [49].

Oxidative stress has been shown to be closely associated with the onset and progression of IDD. Oxidative stress is caused by the excessive accumulation of free radicals and ROS and can damage proteins and nucleic acids, leading to changes in cellular structure and function [50, 51]. The classical antioxidant system comprises the antioxidant enzymes SOD, CAT, GSH-Px, and lipoic acid [52]. As the predominant defensive enzyme in the antioxidative system, SOD catalyzes the disproportionation of superoxide anions, preventing tissue damage [53, 54]. CAT is an additional antioxidant enzyme, catalyzing H_2_O_2_ into H_2_O and O_2_, thus balancing redox reactions [55]. GSH-Px is a powerful free radical scavenger that inhibits lipid peroxidation, while MDA concentration displays the extent of cell membrane lipid peroxidation [56]. Measurements of SOD, CAT, and GSH-Px allow the evaluation of the capability of cells to clear ROS, while MDA reflects the severity of an attack by ROS [57]. Thus, the levels of these indicators in IVDs reflect oxidative stress levels. In the present study, needle puncture, used to establish the model of IDD, resulted in a significant downregulation of SOD, CAT, and GSH-Px activity and increased MDA concentration, thus increasing oxidative stress levels. However, administration of 10 or 20 mg/kg STS inhibited injury-induced oxidative stress in a dose-dependent manner. A number of studies have reported the antioxidant capability of STS, for example, decreased MDA and GSH expression levels in a rat model of stroke [58]. STS was shown to inhibit the production of X-ray induced ROS [59] and ameliorated oxidative stress and lipid metabolism in isoproterenol-induced myocardial infarction [60]. Furthermore, STS suppressed the degree of cardiomyocyte apoptosis via inhibition of phosphorylation of the oxidative stress-related protein JNK [24].

To explore the underlying mechanism by which STS inhibits oxidative stress and IDD, Western blot analysis was utilized to identify changes in pathway-associated protein expression. The p38 MAPK signaling pathway is a branch of the MAPK superfamily, mediating the regulation of various physiological and pathological processes such as inflammation, cell stress, growth, development, and apoptosis [22]. A previous study indicated that a number of signaling pathways and transcript factors including MAPKs, p53, NF-*κ*B, and Nrf2/HO-1 are involved in the regulation of oxidative stress [61]. The current study indicated that needle puncture resulted in the activation of the p38 signaling pathway in the IDD group, while STS ameliorated oxidative stress and IDD in a rat model through inhibition of p38 MAPK. Rannou et al. reported that mechanical overload causes the apoptosis of AF cells by p38 MAPK activation [33]. Blockade of p38 MAPK in cytokine-activated NPCs was shown to reduce the generation of cytokine factors associated with inflammation, pain, and matrix degradation [22]. These *in vitro* studies, in addition to observations in the present study, indicated that STS may reduce oxidative stress and IDD via inhibition of p38 MAPK activity.

There are a number of limitations to the study. Firstly, only animal experiments were used in the evaluation of the effect of STS on IDD. Cell experiments (especially human IVD cells) that investigate the use of a p38 MAPK inhibitor should be further conducted to obtain more convincing results. Secondly, STS receptors and downstream target genes were not explored. Thirdly, although STS exhibited profound anti-inflammatory and antioxidative effects in the present animal study, the potential for treatment and possible complications of STS on human diseases require additional examination through clinical research.

## 5. Conclusion

The present study demonstrated that STS ameliorated injury-induced intervertebral disc degeneration, exerting anti-inflammatory and antioxidative effects in a rat model of IDD, possibly by inhibition of the p38 MAPK signaling pathway.

## Data Availability

No data were used to support this study.
